# Dichlorido(6,6′-dimethyl-2,2′-bipyridine-κ^2^ *N*,*N*′)cobalt(II)

**DOI:** 10.1107/S1600536810036846

**Published:** 2010-09-18

**Authors:** Niloufar Akbarzadeh Torbati, Ali Reza Rezvani, Nasser Safari, Hamideh Saravani, Vahid Amani

**Affiliations:** a Department of Chemistry, University of Sistan and Baluchestan, PO Box 98135-674, Zahedan, Iran; b Department of Chemistry, Shahid Beheshti University, G. C., Evin, Tehran 1983963113, Iran

## Abstract

In the title compound, [CoCl_2_(C_12_H_12_N_2_)], the Co^II^ atom is four-coordinated in a distorted tetra­hedral geometry by two N atoms from a 6,6′-dimethyl-2,2′-bipyridine ligand and two terminal Cl atoms. Inter­molecular C—H⋯Cl hydrogen bonds and π–π stacking inter­actions between the pyridine rings [centroid–centroid distances = 3.788 (1) and 3.957 (1) Å] are present in the crystal structure.

## Related literature

For related structures, see: Akbarzadeh Torbati *et al.* (2010 ▶); Alizadeh *et al.* (2010 ▶); Alizadeh, Kalateh, Ebadi *et al.* (2009 ▶); Alizadeh, Kalateh, Khoshtarkib *et al.* (2009 ▶); Alizadeh, Khoshtarkib *et al.* (2009 ▶); Baker *et al.* (1988 ▶); Itoh *et al.* (2005 ▶); Kou *et al.* (2008 ▶); Onggo *et al.* (2005 ▶).
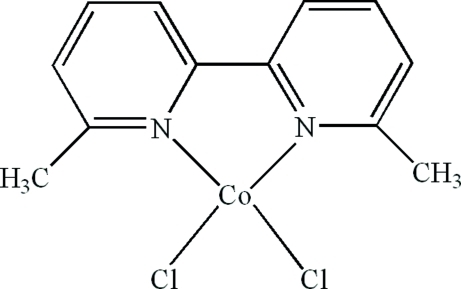


## Experimental

### 

#### Crystal data

[CoCl_2_(C_12_H_12_N_2_)]
*M* *_r_* = 314.07
Monoclinic, 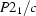

*a* = 7.6292 (14) Å
*b* = 9.8034 (14) Å
*c* = 17.980 (4) Å
β = 93.990 (15)°
*V* = 1341.5 (4) Å^3^
*Z* = 4
Mo *K*α radiation
μ = 1.66 mm^−1^
*T* = 298 K
0.50 × 0.19 × 0.13 mm

#### Data collection

Bruker APEX CCD diffractometer
Absorption correction: multi-scan (*SADABS*; Bruker, 2001 ▶) *T*_min_ = 0.690, *T*_max_ = 0.810
10258 measured reflections
3609 independent reflections
2642 reflections with *I* > 2σ(*I*)
*R*_int_ = 0.100

#### Refinement

*R*[*F* ^2^ > 2σ(*F* ^2^)] = 0.064
*wR*(*F* ^2^) = 0.226
*S* = 1.13
3609 reflections
154 parameters
H-atom parameters constrained
Δρ_max_ = 0.47 e Å^−3^
Δρ_min_ = −0.92 e Å^−3^

### 

Data collection: *SMART* (Bruker, 2007 ▶); cell refinement: *SAINT* (Bruker, 2007 ▶); data reduction: *SAINT*; program(s) used to solve structure: *SHELXTL* (Sheldrick, 2008 ▶); program(s) used to refine structure: *SHELXTL*; molecular graphics: *ORTEP-3* (Farrugia, 1997 ▶); software used to prepare material for publication: *WinGX* (Farrugia, 1999 ▶).

## Supplementary Material

Crystal structure: contains datablocks I, global. DOI: 10.1107/S1600536810036846/hy2353sup1.cif

Structure factors: contains datablocks I. DOI: 10.1107/S1600536810036846/hy2353Isup2.hkl

Additional supplementary materials:  crystallographic information; 3D view; checkCIF report

## Figures and Tables

**Table 1:** Selected bond lengths (Å)

Co1—N1	2.042 (3)
Co1—N2	2.053 (3)
Co1—Cl1	2.2193 (13)
Co1—Cl2	2.2269 (13)

**Table 2:** Hydrogen-bond geometry (Å, °)

*D*—H⋯*A*	*D*—H	H⋯*A*	*D*⋯*A*	*D*—H⋯*A*
C5—H5⋯Cl1^i^	0.93	2.82	3.565 (7)	138

Symmetry code: (i) 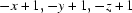.

**Fig. 1.**
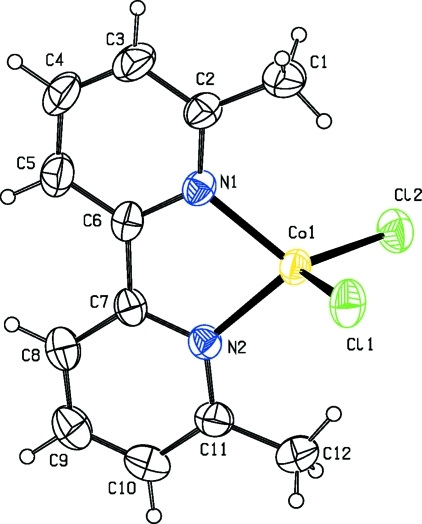
The molecular structure of the title compound. Displacement ellipsoids are drawn at the 30% probability level.

**Fig. 2.**
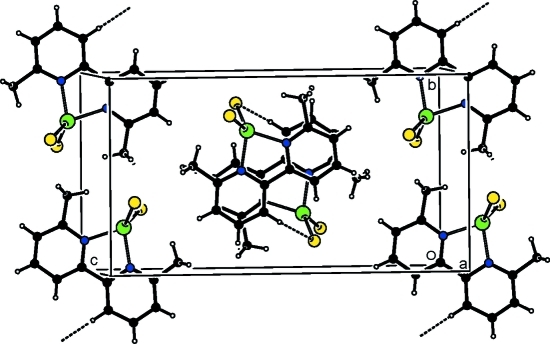
Crystal packing diagram for the title compound. Dashed lines denote hydrogen bonds.

## supplementary crystallographic information

### Comment

6,6'-Dimethyl-2,2'-bipyridine (6,6'-dmbipy) is a good bidentate ligand, and numerous complexes with 6,6'-dmbipy have been prepared, such as that of zinc (Alizadeh, Kalateh, Ebadi *et al.*, 2009; Alizadeh, Kalateh, Khoshtarkib *et al.*, 2009; Alizadeh, Khoshtarkib *et al.*, 2009), copper (Itoh *et al.*, 2005), nickel (Kou *et al.*, 2008), cadmium (Alizadeh *et al.*, 2010) and ruthenium (Onggo *et al.*, 2005). We report here the synthesis and crystal structure of the title compound.

In the title compound (Fig. 1), the Co^II^ atom is four-coordinated in a distorted tetrahedral geometry by two N atoms from a 6,6'-dmbipy ligand and two terminal Cl atoms. The Co—N and Co—Cl bond lengths and angles (Table 1) are within normal range as observed in [Co(6,6'-dmbpy)Cl_2_].1/2(C_6_H_6_) (Baker *et al.*, 1988) and [Co(dmphen)Cl_2_] (dmphen = 2,9-dimethyl-1,10-phenanthroline) (Akbarzadeh Torbati *et al.*, 2010) .

In the crystal structure, intermolecular C—H···Cl hydrogen bonds (Table 2) and π–π contacts (Fig. 2) between the pyridine rings, *Cg*1···*Cg*2^i^ and *Cg*1···*Cg*2^ii^ [symmetry codes: (i) 1-x, 1-y, 1-z; (ii) -x, 1-y, 1-z. *Cg*1 and *Cg*2 are the centroids of the N1, C2–C6 ring and N2, C7–C11 ring], stabilize the structure, with centroid–centroid distances of 3.788 (1) and 3.957 (1) Å.

### Experimental

For the preparation of the title compound, a solution of 6,6'-dmbipy (0.25 g, 1.34 mmol) in methanol (15 ml) was added to a solution of CoCl_2_.6H_2_O (0.37 g, 1.34 mmol) in acetonitrile (15 ml) and the resulting blue solution was stirred for 15 min at 313 K. This solution was left to evaporate slowly at room temperature. After one week, blue block crystals of the title compound were isolated (yield: 0.32 g, 76%).

### Refinement

All H atoms were positioned geometrically and refined as riding atoms, with C—H = 0.93 (aromatic) and 0.96 (methyl) Å and with *U*_iso_(H) = 1.2*U*_eq_(C).

### Crystal data

**Table:**

[CoCl_2_(C_12_H_12_N_2_)]	*F*(000) = 636
*M**_r_* = 314.07	*D*_x_ = 1.555 Mg m^−^^3^
Monoclinic, *P*2_1_/*c*	Mo *K*α radiation, λ = 0.71073 Å
Hall symbol: -P 2ybc	Cell parameters from 1009 reflections
*a* = 7.6292 (14) Å	θ = 2.3–29.3°
*b* = 9.8034 (14) Å	µ = 1.66 mm^−^^1^
*c* = 17.980 (4) Å	*T* = 298 K
β = 93.990 (15)°	Block, blue
*V* = 1341.5 (4) Å^3^	0.50 × 0.19 × 0.13 mm
*Z* = 4	

### Data collection

**Table:**

Bruker APEX CCD diffractometer	3609 independent reflections
Radiation source: fine-focus sealed tube	2642 reflections with *I* > 2σ(*I*)
graphite	*R*_int_ = 0.100
φ and ω scans	θ_max_ = 29.3°, θ_min_ = 2.3°
Absorption correction: multi-scan (*SADABS*; Bruker, 2001)	*h* = −10→10
*T*_min_ = 0.690, *T*_max_ = 0.810	*k* = −13→11
10258 measured reflections	*l* = −24→24

### Refinement

**Table:**

Refinement on *F*^2^	Primary atom site location: structure-invariant direct methods
Least-squares matrix: full	Secondary atom site location: difference Fourier map
*R*[*F*^2^ > 2σ(*F*^2^)] = 0.064	Hydrogen site location: inferred from neighbouring sites
*wR*(*F*^2^) = 0.226	H-atom parameters constrained
*S* = 1.13	*w* = 1/[σ^2^(*F*_o_^2^) + (0.1251*P*)^2^ + 0.225*P*] where *P* = (*F*_o_^2^ + 2*F*_c_^2^)/3
3609 reflections	(Δ/σ)_max_ < 0.001
154 parameters	Δρ_max_ = 0.47 e Å^−^^3^
0 restraints	Δρ_min_ = −0.92 e Å^−^^3^

### Fractional atomic coordinates and isotropic or equivalent isotropic displacement parameters (Å^2^)

**Table:**

	*x*	*y*	*z*	*U*_iso_*/*U*_eq_	
C1	0.3248 (9)	0.4581 (6)	0.2579 (3)	0.0900 (16)	
H1A	0.4369	0.4668	0.2373	0.108*	
H1B	0.2338	0.4866	0.2217	0.108*	
H1C	0.3061	0.3646	0.2712	0.108*	
C2	0.3209 (6)	0.5449 (5)	0.3254 (3)	0.0654 (10)	
C3	0.3451 (8)	0.6844 (6)	0.3227 (4)	0.0832 (15)	
H3	0.3656	0.7264	0.2777	0.100*	
C4	0.3390 (8)	0.7594 (5)	0.3849 (4)	0.0857 (16)	
H4	0.3592	0.8529	0.3835	0.103*	
C5	0.3022 (7)	0.6964 (5)	0.4517 (4)	0.0778 (14)	
H5	0.2945	0.7475	0.4950	0.093*	
C6	0.2773 (5)	0.5568 (4)	0.4525 (2)	0.0572 (9)	
C7	0.2356 (5)	0.4790 (4)	0.5193 (2)	0.0544 (8)	
C8	0.2233 (6)	0.5387 (6)	0.5874 (3)	0.0719 (12)	
H8	0.2462	0.6312	0.5940	0.086*	
C9	0.1762 (7)	0.4593 (7)	0.6459 (3)	0.0807 (14)	
H9	0.1637	0.4985	0.6924	0.097*	
C10	0.1481 (7)	0.3233 (7)	0.6357 (3)	0.0765 (13)	
H10	0.1156	0.2693	0.6749	0.092*	
C11	0.1681 (7)	0.2654 (5)	0.5660 (3)	0.0661 (10)	
C12	0.1486 (11)	0.1176 (6)	0.5507 (4)	0.100 (2)	
H12A	0.0319	0.0892	0.5603	0.120*	
H12B	0.2324	0.0678	0.5824	0.120*	
H12C	0.1688	0.1000	0.4995	0.120*	
N1	0.2875 (4)	0.4836 (3)	0.38883 (19)	0.0537 (7)	
N2	0.2096 (4)	0.3440 (4)	0.50901 (18)	0.0541 (7)	
Co1	0.24226 (7)	0.28007 (5)	0.40212 (3)	0.0549 (2)	
Cl1	0.47387 (17)	0.14706 (13)	0.38988 (8)	0.0811 (4)	
Cl2	0.01041 (17)	0.19582 (14)	0.33528 (8)	0.0769 (4)	

### Atomic displacement parameters (Å^2^)

**Table:**

	*U*^11^	*U*^22^	*U*^33^	*U*^12^	*U*^13^	*U*^23^
C1	0.124 (4)	0.079 (3)	0.071 (3)	−0.003 (3)	0.029 (3)	0.007 (3)
C2	0.068 (2)	0.058 (2)	0.071 (3)	−0.0068 (19)	0.0113 (19)	0.0074 (19)
C3	0.089 (3)	0.064 (3)	0.097 (4)	−0.021 (3)	0.006 (3)	0.021 (3)
C4	0.096 (4)	0.049 (2)	0.111 (5)	−0.018 (2)	0.003 (3)	0.005 (3)
C5	0.084 (3)	0.058 (2)	0.090 (3)	−0.010 (2)	−0.006 (3)	−0.013 (2)
C6	0.0507 (17)	0.0491 (19)	0.072 (2)	0.0005 (15)	0.0021 (16)	−0.0091 (17)
C7	0.0490 (17)	0.059 (2)	0.0559 (19)	0.0040 (15)	0.0048 (14)	−0.0108 (16)
C8	0.075 (3)	0.074 (3)	0.068 (3)	0.002 (2)	0.010 (2)	−0.019 (2)
C9	0.082 (3)	0.095 (4)	0.066 (3)	0.009 (3)	0.011 (2)	−0.018 (3)
C10	0.073 (3)	0.099 (4)	0.058 (2)	0.006 (3)	0.009 (2)	0.010 (2)
C11	0.073 (3)	0.069 (3)	0.057 (2)	0.001 (2)	0.0055 (18)	0.0050 (19)
C12	0.154 (6)	0.071 (3)	0.077 (3)	−0.019 (4)	0.017 (4)	0.014 (3)
N1	0.0525 (15)	0.0479 (16)	0.0617 (17)	−0.0034 (13)	0.0120 (13)	−0.0002 (13)
N2	0.0572 (16)	0.0543 (17)	0.0512 (16)	0.0003 (14)	0.0072 (13)	−0.0035 (13)
Co1	0.0631 (4)	0.0453 (3)	0.0569 (4)	−0.0020 (2)	0.0095 (2)	−0.0053 (2)
Cl1	0.0798 (7)	0.0659 (7)	0.0979 (9)	0.0142 (6)	0.0080 (6)	−0.0239 (6)
Cl2	0.0753 (7)	0.0790 (7)	0.0759 (7)	−0.0148 (6)	0.0013 (5)	−0.0134 (6)

### Geometric parameters (Å, °)

**Table:**

C1—C2	1.483 (7)	C8—C9	1.377 (8)
C1—H1A	0.9600	C8—H8	0.9300
C1—H1B	0.9600	C9—C10	1.361 (9)
C1—H1C	0.9600	C9—H9	0.9300
C2—N1	1.330 (5)	C10—C11	1.393 (7)
C2—C3	1.382 (7)	C10—H10	0.9300
C3—C4	1.342 (9)	C11—N2	1.338 (6)
C3—H3	0.9300	C11—C12	1.480 (8)
C4—C5	1.397 (9)	C12—H12A	0.9600
C4—H4	0.9300	C12—H12B	0.9600
C5—C6	1.382 (6)	C12—H12C	0.9600
C5—H5	0.9300	Co1—N1	2.042 (3)
C6—N1	1.358 (5)	Co1—N2	2.053 (3)
C6—C7	1.477 (6)	Co1—Cl1	2.2193 (13)
C7—N2	1.349 (5)	Co1—Cl2	2.2269 (13)
C7—C8	1.365 (5)		
			
C2—C1—H1A	109.5	C10—C9—C8	119.8 (5)
C2—C1—H1B	109.5	C10—C9—H9	120.1
H1A—C1—H1B	109.5	C8—C9—H9	120.1
C2—C1—H1C	109.5	C9—C10—C11	119.7 (5)
H1A—C1—H1C	109.5	C9—C10—H10	120.2
H1B—C1—H1C	109.5	C11—C10—H10	120.2
N1—C2—C3	120.7 (5)	N2—C11—C10	120.0 (5)
N1—C2—C1	117.3 (4)	N2—C11—C12	116.6 (4)
C3—C2—C1	121.9 (5)	C10—C11—C12	123.4 (5)
C4—C3—C2	120.0 (5)	C11—C12—H12A	109.5
C4—C3—H3	120.0	C11—C12—H12B	109.5
C2—C3—H3	120.0	H12A—C12—H12B	109.5
C3—C4—C5	119.7 (5)	C11—C12—H12C	109.5
C3—C4—H4	120.1	H12A—C12—H12C	109.5
C5—C4—H4	120.1	H12B—C12—H12C	109.5
C6—C5—C4	118.8 (5)	C2—N1—C6	120.7 (4)
C6—C5—H5	120.6	C2—N1—Co1	125.8 (3)
C4—C5—H5	120.6	C6—N1—Co1	113.5 (3)
N1—C6—C5	120.0 (5)	C11—N2—C7	120.0 (4)
N1—C6—C7	116.2 (3)	C11—N2—Co1	126.3 (3)
C5—C6—C7	123.9 (4)	C7—N2—Co1	113.7 (3)
N2—C7—C8	121.6 (4)	N1—Co1—N2	81.00 (14)
N2—C7—C6	115.7 (3)	N1—Co1—Cl1	114.87 (10)
C8—C7—C6	122.7 (4)	N2—Co1—Cl1	114.93 (10)
C7—C8—C9	118.8 (5)	N1—Co1—Cl2	115.70 (10)
C7—C8—H8	120.6	N2—Co1—Cl2	118.35 (10)
C9—C8—H8	120.6	Cl1—Co1—Cl2	109.67 (5)
			
N1—C2—C3—C4	1.6 (8)	C5—C6—N1—Co1	−179.0 (3)
C1—C2—C3—C4	179.4 (6)	C7—C6—N1—Co1	−0.2 (4)
C2—C3—C4—C5	−2.2 (9)	C10—C11—N2—C7	1.3 (7)
C3—C4—C5—C6	1.7 (9)	C12—C11—N2—C7	−177.7 (5)
C4—C5—C6—N1	−0.5 (7)	C10—C11—N2—Co1	−179.2 (4)
C4—C5—C6—C7	−179.2 (5)	C12—C11—N2—Co1	1.7 (7)
N1—C6—C7—N2	−1.0 (5)	C8—C7—N2—C11	1.0 (6)
C5—C6—C7—N2	177.7 (4)	C6—C7—N2—C11	−178.9 (4)
N1—C6—C7—C8	179.2 (4)	C8—C7—N2—Co1	−178.5 (3)
C5—C6—C7—C8	−2.1 (6)	C6—C7—N2—Co1	1.6 (4)
N2—C7—C8—C9	−2.6 (7)	C2—N1—Co1—N2	−178.0 (3)
C6—C7—C8—C9	177.2 (4)	C6—N1—Co1—N2	0.8 (3)
C7—C8—C9—C10	1.9 (8)	C2—N1—Co1—Cl1	68.6 (3)
C8—C9—C10—C11	0.4 (8)	C6—N1—Co1—Cl1	−112.6 (2)
C9—C10—C11—N2	−2.0 (8)	C2—N1—Co1—Cl2	−60.8 (3)
C9—C10—C11—C12	177.0 (6)	C6—N1—Co1—Cl2	118.0 (2)
C3—C2—N1—C6	−0.4 (7)	C11—N2—Co1—N1	179.2 (4)
C1—C2—N1—C6	−178.3 (4)	C7—N2—Co1—N1	−1.4 (3)
C3—C2—N1—Co1	178.3 (4)	C11—N2—Co1—Cl1	−67.5 (4)
C1—C2—N1—Co1	0.4 (6)	C7—N2—Co1—Cl1	111.9 (3)
C5—C6—N1—C2	−0.1 (6)	C11—N2—Co1—Cl2	64.7 (4)
C7—C6—N1—C2	178.7 (3)	C7—N2—Co1—Cl2	−115.8 (2)

### Hydrogen-bond geometry (Å, °)

**Table:**

*D*—H···*A*	*D*—H	H···*A*	*D*···*A*	*D*—H···*A*
C5—H5···Cl1^i^	0.93	2.82	3.565 (7)	138

Symmetry codes: (i) −*x*+1, −*y*+1, −*z*+1.
